# Evaluation of ovarian stiffness and its biological mechanism using shear wave elastography in polycystic ovary syndrome

**DOI:** 10.1038/s41598-024-84338-8

**Published:** 2025-01-02

**Authors:** Yifang He, Shuangping Deng, Yanli Wang, Xiali Wang, Qingqing Huang, Jing Cheng, Dandan Wang, Guorong Lyu

**Affiliations:** 1 Department of Ultrasound, The Second Affiliated Hospital of Fujian Medical University, No. 34 North Zhongshan Road, Quanzhou, 362000 Fujian Province China; 2 Quanzhou Medical College, Quanzhou, China

**Keywords:** Polycystic ovary syndrome, Shear wave elastography, Ovarian stiffness, Fibrosis, Basement membranes, Endocrine reproductive disorders, Imaging techniques

## Abstract

Polycystic ovary syndrome (PCOS) is a common endocrine disorder with various contributing factors. Shear wave elastography (SWE) is a contemporary noninvasive imaging technique that reports on the elasticity of tissues. This study aimed to evaluate ovarian stiffness in patients with PCOS using transvaginal SWE, and investigate the potential biological mechanisms underlying increased ovarian stiffness. Patients with PCOS and healthy controls underwent transvaginal 2D ultrasound and SWE to measure the number of follicles, ovarian volume, and ovarian elasticity. Multivariate logistic regression analysis was conducted to identify risk factors for PCOS. A rat model of PCOS was established to further investigate the biological basis of increased ovarian stiffness. Histological analysis, enzyme-linked immunosorbent assay, quantitative reverse transcription-polymerase chain reaction, western blotting, transcriptomics, and proteomics were performed to assess alterations in fibrosis and basement membrane (BM) gene expression. The results demonstrated that patients with PCOS (*n* = 59) showed an increased number of follicles, ovarian volume, and SWE (mean and max) compared with controls (*n* = 56; *P* < 0.001). The number of follicles, ovarian volume, and SWE_mean were identified as independent risk factors for PCOS (*P* < 0.05). SWE_mean ≥ 12.5 kPa demonstrated an area under the curve of 0.816 for PCOS diagnosis and was positively correlated with AMH levels (*r* = 0.6776, *P* < 0.0001). In the rat model, increased ovarian stiffness was associated with significant fibrosis and altered expression of fibrosis-related markers. Transcriptomic and proteomic analyses revealed that BM gene alterations were correlated with ovarian stiffness, which was further validated using PCOS patient data from the Gene Expression Omnibus database. In conclusion, SWE is a valuable technique for diagnosing PCOS by detecting increased ovarian stiffness, which may be associated with alterations in the expression of BMs, thereby mediating ovarian fibrosis.

**Supplementary Information:**

The online version contains supplementary material available at 10.1038/s41598-024-84338-8.

## Introduction

Polycystic ovary syndrome (PCOS) is the most prevalent endocrine disorder affecting women of reproductive age, with a global prevalence ranging from 5–20%^1^. It is characterized by a wide range of symptoms, including hyperandrogenism, ovulatory dysfunction, and polycystic ovarian morphology. In addition, PCOS is frequently associated with metabolic disorders, such as insulin resistance, obesity, and an elevated risk of type 2 diabetes mellitus and cardiovascular disease^2–4^. Diagnosing PCOS at an early stage is crucial for establishing a focused therapeutic intervention that can greatly improve patient outcomes and prognoses.

Based on the 2003 Rotterdam Conference, the diagnosis of PCOS is confirmed when two or more of the following conditions are met: (1) oligomenorrhea or amenorrhea, (2) hyperandrogenemia with clinical or biochemical signs, and (3) polycystic ovarian morphology as observed via ultrasonography^3,5^. The third criterion relies on ovarian morphology for diagnosis; however, the polycystic acoustic pattern is not unique to PCOS and can appear in other conditions; hence, there are still a lack of diagnostic techniques to fully characterize the lesions in patients with PCOS. Conventional ultrasound examinations usually have difficulty presenting the overall morphology and structure of the ovary in patients with PCOS, and physicians can only observe follicles in a limited section of the ovary, complicating the process of follicle counting. The subjective judgment of the operating physician can easily result in misdiagnosis. Therefore, the development of more efficient and sensitive diagnostic methods is necessary.

Ultrasound elastography (UE) is an emerging imaging technique that can be used to image tissues with different degrees of elasticity and deformation following the application of an external force. It provides diagnostic information regarding tissue stiffness and complements traditional ultrasound assessments of morphology and blood flow characteristics^6^. Shear wave elastography (SWE) creates transverse waves through emitted acoustic radiation pulses that induce tissue vibration. Color-coding technology is then used to visualize tissue elasticity in real time and calculate the Young’s modulus, with higher values indicating greater tissue stiffness^7,8^. The SWE technique has been applied clinically, including in the assessment of liver fibrosis^9^, thyroid nodules^10^, and breast lesions^11^, and is considered appropriate for obstetrics and gynecology^12^. The main diagnostic criteria for PCOS are ovarian morphology and function, and SWE is a valuable diagnostic tool for measuring changes in ovarian stiffness.

Recent studies have shown that increased ovarian stiffness measured by UE may be a significant characteristic of PCOS^13,14^. However, there are currently no relevant reports on the changes in ovarian stiffness as detected by transvaginal ultrasound SWE in patients with PCOS, and there are no further studies on the mechanism of changes in ultrasound parameters in PCOS. In this study, we aimed to evaluate ovarian stiffness in women with PCOS using transvaginal SWE and investigate the underlying biological mechanisms using a rat model of PCOS. By combining imaging with histological and molecular data analysis, we not only validated SWE as a diagnostic tool for PCOS but also explored the molecular foundation behind the images in-depth, which could contribute to the development of diagnostic and therapeutic strategies for this disease.

## Methods

### Study design and ethical approval

This study involved human participation in clinical investigations and animal models in experimental studies. The study complied with the Declaration of Helsinki and was approved by the Institutional Review Board of the Second Affiliated Hospital of Fujian Medical University (No. 2023357). All participating women provided written informed consent. The animal care and experiments complied with the guidelines of the Chinese Animal Experimentation Committee and were approved by the Laboratory Animal Institutional Review Committee of Quanzhou Medical College (No. 2024017).

### Clinical study

#### Participants

This prospective study recruited participants between April and October 2023 from the Department of Ultrasound, Second Affiliated Hospital of Fujian Medical University. The study included women diagnosed with PCOS based on the Rotterdam Criteria. The control group comprised of age-matched healthy women with regular menstrual cycles, no clinical signs of hyperandrogenism, and normal ovarian morphology on ultrasonography. The exclusion criteria were abnormal thyroid function, type 2 diabetes mellitus, Cushing’s syndrome, congenital adrenocortical hyperplasia, hyperprolactinemia, premature ovarian failure, recent medication use (e.g., birth control pills and glucocorticoids), follicle diameter > 9 mm, ovarian tumors, and unwillingness to participate. Age, body mass index (BMI), sex hormone levels, and ultrasound parameters (collected on days 3–5 of the menstrual cycle) were recorded (Table 1).

#### Transvaginal 2D ultrasound and SWE examination

**Fig. 1:**
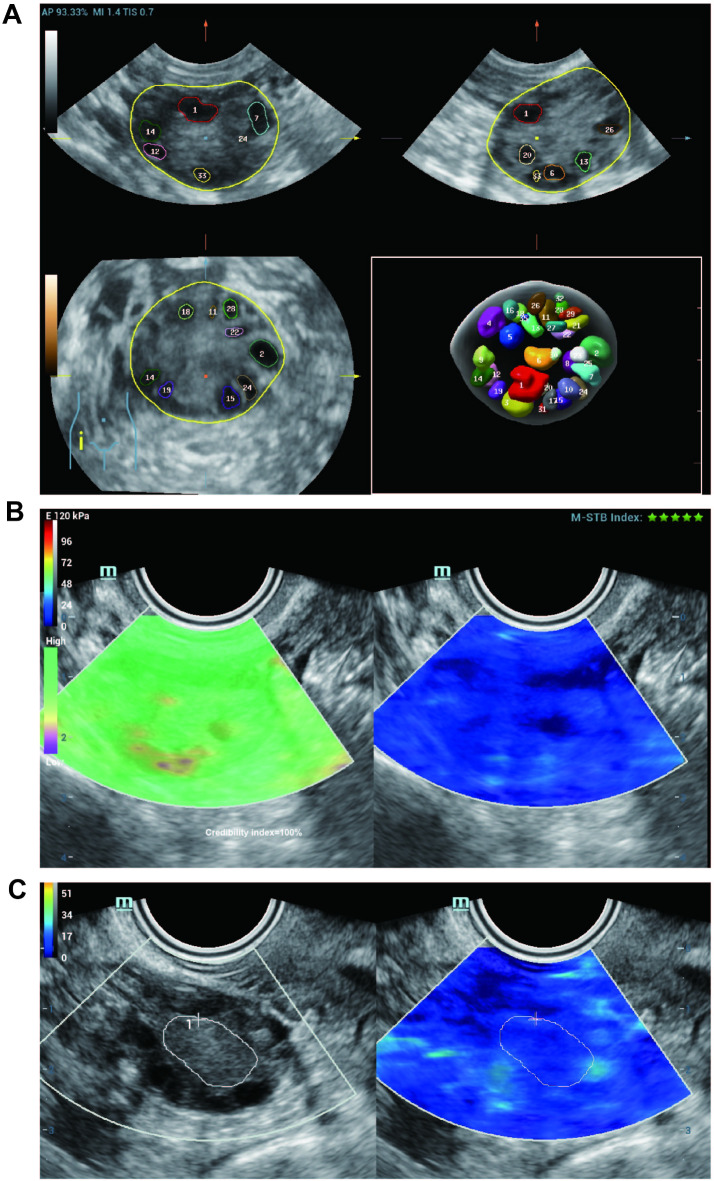
Schematic diagram of measurement of human sinus follicle number and ovarian elasticity values. (**A**) A schematic diagram of sinus follicle counts obtained using SonoAVC automated measurement software; (**B**, **C**) Schematic diagram of ovarian medulla elasticity value measurements. All SWE measurements were performed in split-screen mode. Green left screen sampling box indicates high confidence, while purple color indicates low confidence. Blue in the right screen sampling box indicates soft tissue, and red indicates hard tissue. The region of interest is manually outlined ovarian medulla contours.

Each participant underwent transvaginal ultrasonography using a Mindray color Doppler system (model NuewaR9, probe model DE10-3WU, frequency range: 3–12 MHz) from Mindray, Shenzhen, China. A sonographer proficient in gynecological ultrasonography conducted all ultrasound scans. The physician autonomously performed the procedure without prior knowledge of the medical backgrounds or clinical states of the patients. Ovarian evaluation was performed during the early follicular phase (days 3–5) of the menstrual cycle. Participants emptied their bladders and assumed the lithotomy position. Measurements of the uterus and ovaries included length, width, thickness, and volume (volume [cm³] = length × width × thickness × 0.5236)^15^, as well as endometrial thickness and the maximal diameter of the largest follicle. Antral follicle counts were determined using VOCAL technology in conjunction with SonoAVC software (Fig. 1A). Subsequently, the elastography mode was initiated, necessitating the participants to hold their breath and be still for 3–5 s to stabilize the elastography process, which minimizes the elastic distribution differences between the deep and superficial layers, ensuring a uniform image and maintaining the Q-box within 10 mm. Following stabilization, the ovarian medulla was delineated manually (Figs. 1B and C), and the mean (SWE_mean), maximum (SWE_max), and minimum (SWE_min) Young’s moduli were measured in kilopascals (kPa). Three measurements were obtained and averaged for each region, and the results for both ovaries were averaged.

#### Data analysis

Statistical analyses were conducted with GraphPad Prism 9 and SPSS Statistics 26. Normally distributed measurements were reported as mean ± standard deviation (SD). Independent sample t-tests were employed to compare the groups. The intraclass correlation coefficient (ICC) and Bland–Altman test were used to assess the reliability and consistency of repeated measurements taken by the same and different physicians. For an ICC between 0 and 1, an ICC closer to 1 indicated better reproducibility and less measurement error. Multivariate analyses of ultrasound parameters and PCOS outcomes were performed using a variance inflation factor (VIF) > 10 to assess covariance. The results were presented as odds ratios (OR) with 95% confidence intervals. Receiver operating characteristic (ROC) curves were used to assess the diagnostic efficacy of the follicle count, ovarian volume, and mean SWE for PCOS. The correlation between SWE_mean and anti-Müllerian tubular hormone (AMH) levels was assessed using Pearson’s correlation analysis. Statistical significance was set at *P* < 0.05.

### Animal study

All methods are reported in accordance with ARRIVE guidelines^16^.

#### Construction of the PCOS rat model

The rats were purchased from Fuzhou Houji Laboratory Animal Center. All animals were housed in specific pathogen free (SPF) grade plastic cages with a 12 h light/dark cycle, temperature of 20–24 °C, 50% humidity, and free access to sterilized food and water. Bedding for these animals was changed twice a week. Twenty-four 3-week-old (SD, 55 ± 3 g) female Sprague-Dawley rats were randomly divided into two groups: PCOS group (*n* = 12) and control group (*n* = 12). To induce PCOS, dehydroepiandrosterone (DHEA) (Shanghai Yuanye Biotechnology Co., LTD, Shanghai, China) (6 mg/100 g) was dissolved in 0.2 ml of sesame oil (Yuanye, Shanghai, China) according to the body weight of the rats and injected subcutaneously on the back of the neck for  21 consecutive days. The control group received an equivalent volume of sesame oil without DHEA.

In the final 7 days of the modeling phase, 6 rats per group were randomly chosen for vaginal secretion collection via cytology at 9:00 AM. Rats were injected intraperitoneally with sodium pentobarbital, after which blood samples were obtained by cardiac puncture and centrifuged, and the serum was frozen at -80 °C. The blood samples were then stored in liquid nitrogen. The ovarian tissue was divided and fixed with 4% paraformaldehyde on one side and stored in liquid nitrogen on the other side. The rats were then euthanized with sodium pentobarbital. Hematoxylin and eosin staining, sex hormone level testing, emotional cycle assessment, and Masson’s trichrome staining were performed (Supplemental Methods).

#### Transabdominal 2D ultrasound and SWE examination in rats

**Fig. 2:**
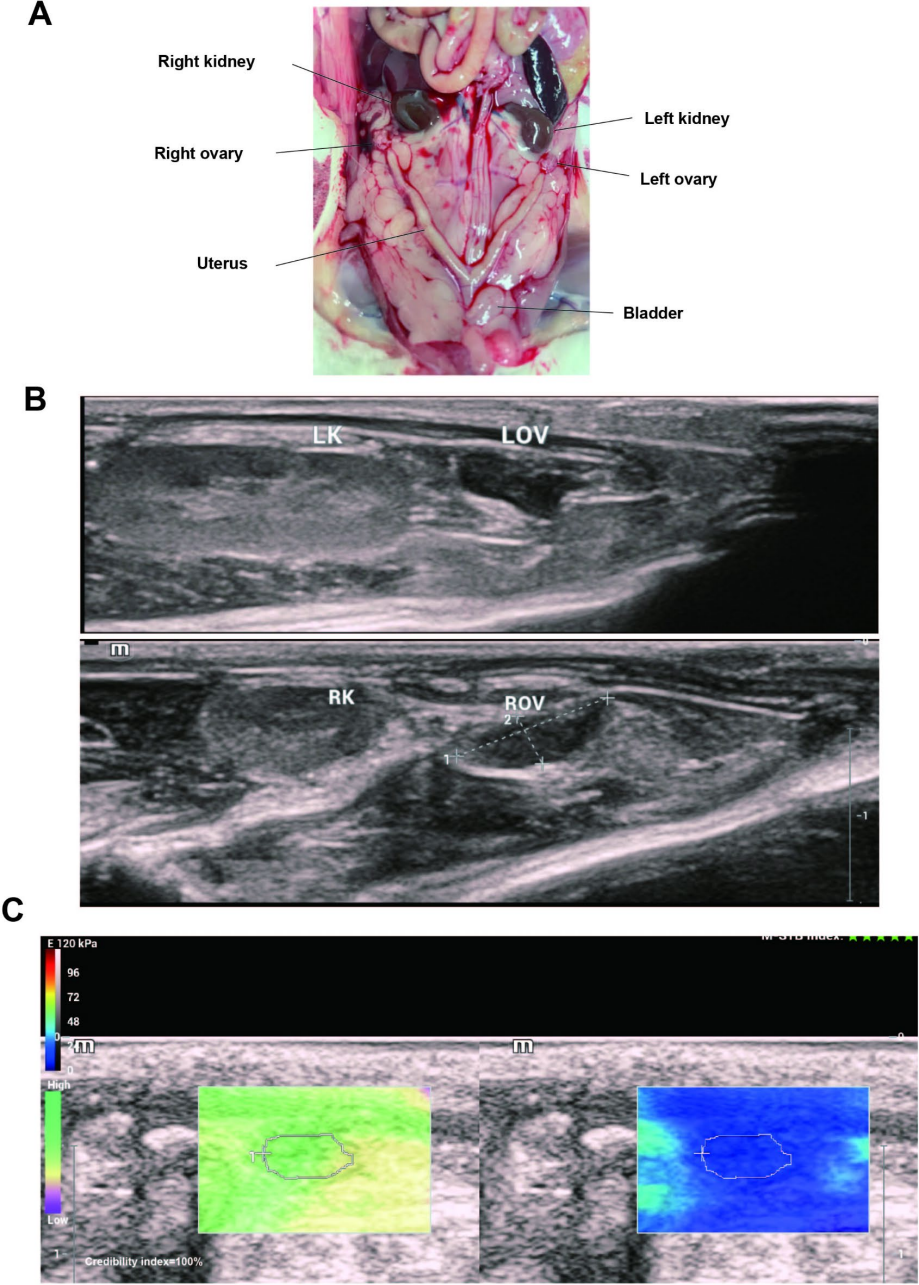
Schematic diagram of ovaries measured by 2-D ultrasound and SWE in rats. (**A**) Schematic diagram of rat anatomy; (**B**) Schematic diagram of the ovary in the rat with 2-D ultrasound; (**C**) Schematic diagram depicting the measurement of ovarian elasticity values in rats. All SWE measurements were performed in split-screen mode. Green left screen sampling box indicates high confidence, while purple color indicates low confidence. Blue in the right screen sampling box indicates soft tissue, and red indicates hard tissue. The region of interest is manually outlined ovarian contours. LK, left kidney; RK, right kidney; LOV, left ovary; ROV, right ovary.

The rats were subjected to ultrasonography using a Mindray color Doppler ultrasound system (model Resona I9S, probe model L14-3WS; Mindray, Shenzhen, China) at a frequency of 7.5–14.5 MHz. An acoustic gel was applied to the transducer and directly coupled to rat skin. A cross-sectional scan was performed using the bladder as a marker to locate the cervical and uterine horn branches, followed by a longitudinal scan displaying the entire uterus on one side. The ovaries were located at the ends of the uterus, and the kidneys were located above them. The length, width, and thickness of the ovaries were measured, and their volumes were subsequently calculated. After the high-frequency ultrasound scan, the device was switched to the elastography mode, and the rats were kept stable for 3–5 s to ensure a uniform image with a maximum O-box of 5 mm. The contours of the ovaries were outlined manually, and the Young’s moduli of the rat ovaries were calculated as the mean (SWE_mean), maximum (SWE_max), and minimum (SWE_min) values (Fig. 2). Three measurements were taken at each site and averaged for each ovary.

#### Molecular analysis

The levels of fibrosis markers were examined in the rat models. Serum connective tissue growth factor (CTGF) levels were measured using enzyme-linked immunosorbent assay, and the mRNA expression of Lamc3 and Col4a1 in ovarian tissues was assessed using quantitative reverse transcription-polymerase chain reaction. The TGF-β1, α-SMA, and Col3a protein levels in ovarian tissues were analyzed using western blotting (Supplemental Methods).

#### RNA-sequencing (RNA-seq) and proteomics

Three rats each from the PCOS and control groups were randomly selected for RNA-seq and proteomic analyses of ovarian tissue. The total RNA was extracted using the TRIzol reagent (Invitrogen, Carlsbad, CA, USA), and libraries were sequenced on an Illumina NovaSeq platform. Differentially expressed genes (DEGs) were identified with |log_2_ (fold-change) | >1 and an adjusted *P*-value of < 0.05.

Proteins were extracted with an SDT buffer, and trypsin digestion was carried out following the filter-aided sample preparation method described by Wiśniewski et al.^17^. The peptide content was quantified at a wavelength of 280 nm. The LC-MS/MS analysis employed data-independent acquisition (DIA) with an astral mass spectrometer (Thermo Scientific)^18^. Differentially expressed proteins (DEPs) were identified with |log_2_ (fold change) | >0.585 and *P* < 0.05 (Supplemental Methods).

#### Bioinformatics analysis

We validated our findings in the rat model by reanalyzing three Gene Expression Omnibus (GEO) datasets, namely GSE137684, GSE34526, and GSE5850, which included ovarian granulosa cell (GC) samples from 21 patients with PCOS and 13 controls. We assessed the differential expression of basement membrane (BM) genes and performed Gene Ontology (GO) functional analyses, Kyoto Encyclopedia of Genes and Genomes (KEGG) pathway analyses, and immune infiltration scoring to investigate the role of BM genes in PCOS development (Supplemental Methods).

## Results

### The SWE suggested an increased ovarian stiffness in patients with PCOS

**Fig. 3:**
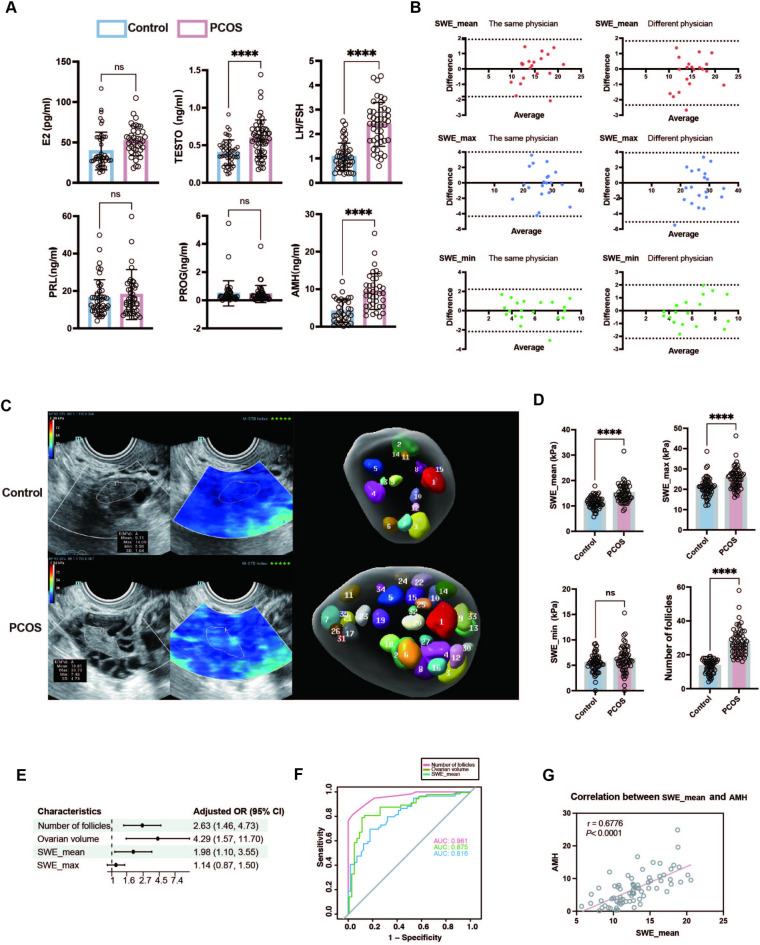
Clinical and ultrasound parameters and related diagnostic and regression analyses in patients with PCOS. (**A**) Serum levels of sex hormones in PCOS patients and healthy controls; (**B**) Bland-Altman plot of SWE measuring ovarian medulla by different and the same sonographers (*n* = 20); (**C**,** D**) Representative images of Young’s modulus of the ovarian medulla and sinus follicle counts in PCOS patient and healthy control and corresponding histogram statistics; (**E**) Multivariate regression analysis used to predict PCOS and visualized using a forest plot; (**F**) A ROC curve was plotted to evaluate the diagnostic value of number of follicles, ovarian volume, and SWE_mean in PCOS; (**G**) Correlation between SWE_ mean and AMH levels. PRGE, progesterone; PRL, prolactin; E2, estradiol, FSH, follicle-stimulating hormone; LH, luteinizing hormone; TESTO, testosterone; AMH, anti-Müllerian tubular hormone; SWE, shear wave elastography; OR, odds ratio OR; CI, confidence interval. ****P < 0.0001, ns, not significant.

The clinical, anthropometric, and laboratory characteristics of the participants are presented in Table 1. Patients with PCOS (*n* = 59) had significantly higher BMI (22.29 ± 1.26 vs. 23.12 ± 1.06), TESTO (0.40 ± 0.17 vs. 0.60 ± 0.24), LH/FSH (1.07 ± 0.56 vs. 2.39 ± 0.90), and AMH (4.2 ± 3.0 vs. 9.4 ± 4.8) than the control group (*n* = 56; *P* < 0.001; Fig. 3A). The ICC for the SWE_mean, SWE_max, and SWE_min of the ovaries measured by the same and different physicians were all > 0.8, indicating excellent repeatability (Table S2). The Bland–Altman analysis revealed that over 95% of the data points from the same and different physicians fell within the 95% consistency threshold, indicating good agreement (Fig. 3B**)**.

**Table 1:** Demographics and baseline characteristics of the patients.

Characteristic	Group		t	*P*
	Control, *N* = 56	PCOS, *N* = 59		
Age	31.0 ± 4.9	29.4 ± 4.5	1.91	0.059
BMI	22.29 ± 1.26	23.12 ± 1.06	-3.82	< 0.001
E2	45 ± 27	50 ± 17	-0.86	0.391
TESTO	0.40 ± 0.17	0.60 ± 0.24	-4.83	< 0.001
LH/FSH	1.07 ± 0.56	2.39 ± 0.90	-8.5	< 0.001
PRL	16 ± 10	18 ± 13	-0.74	0.46
PROG	0.76 ± 2.05	0.68 ± 1.69	0.21	0.835
AMH	4.2 ± 3.0	9.4 ± 4.8	-5.64	< 0.001

Abbreviations: BMI, body mass index; E2, estradiol; TESTO, testosterone; LH, luteinizing hormone; FSH, follicle-stimulating hormone; PRL, prolactin; PROG, progesterone; AMH, anti-Müllerian tubular hormone.

The number of follicles (14 ± 4 vs. 26 ± 8), ovarian volume (5.80 ± 1.23 vs. 8.10 ± 1.48 cm^3^), SWE_mean (11.4 ± 2.3 vs. 14.9 ± 3.6 kPa), and SWE_max (21.1 ± 4.6 vs. 25.4 ± 5.2 kPa) as measured by 2D ultrasound. In addition, SWE were significantly higher in the ovarian medulla of patients with PCOS compared to controls (*P* < 0.001). The analysis of covariance showed that the VIFs for the four factors were < 10 (1.495, 1.378, 1.404, and 1.178, respectively). Multivariate regression analysis showed that the number of follicles, ovarian volume, and SWE_mean were independent risk factors for PCOS (number of follicles, OR = 2.63, *P* = 0.001; ovarian volume, OR = 4.29, *P* = 0.004; SWE_mean, OR = 1.98, *P* = 0.023; Figs. 3C -E ; Tables 2, S3, and S4**)**.

**Table 2:** Multivariate regression analysis used to predict PCOS.

Characteristic	OR	95% CI	*P*
Number of follicles	2.63	1.46, 4.73	0.001
Ovarian volume	4.29	1.57, 11.70	0.004
SWE_mean	1.98	1.10, 3.55	0.023
SWE_max	1.14	0.87, 1.50	0.348

Abbreviations: OR, Odds Ratios; CI, Confidence Interval.

The ROC curve analysis yielded area under the curve values of 0.961, 0.875, and 0.816 for the number of follicles (cutoff of 18), ovarian volume (cutoff of 7.155), and SWE_mean (cutoff of 12.5), respectively (Fig. 3F). The sensitivity, specificity, positive predictive value, and negative predictive value for PCOS diagnosis were shown in Table S5. In addition, the mean SWE index of UE was positively correlated with AMH levels (*r* = 0.6776, *P* < 0.0001; Fig. 3G), which suggested that increased ovarian stiffness in PCOS may be associated with higher AMH levels.

### Ovarian stiffness increased in the rat model of PCOS

**Fig. 4:**
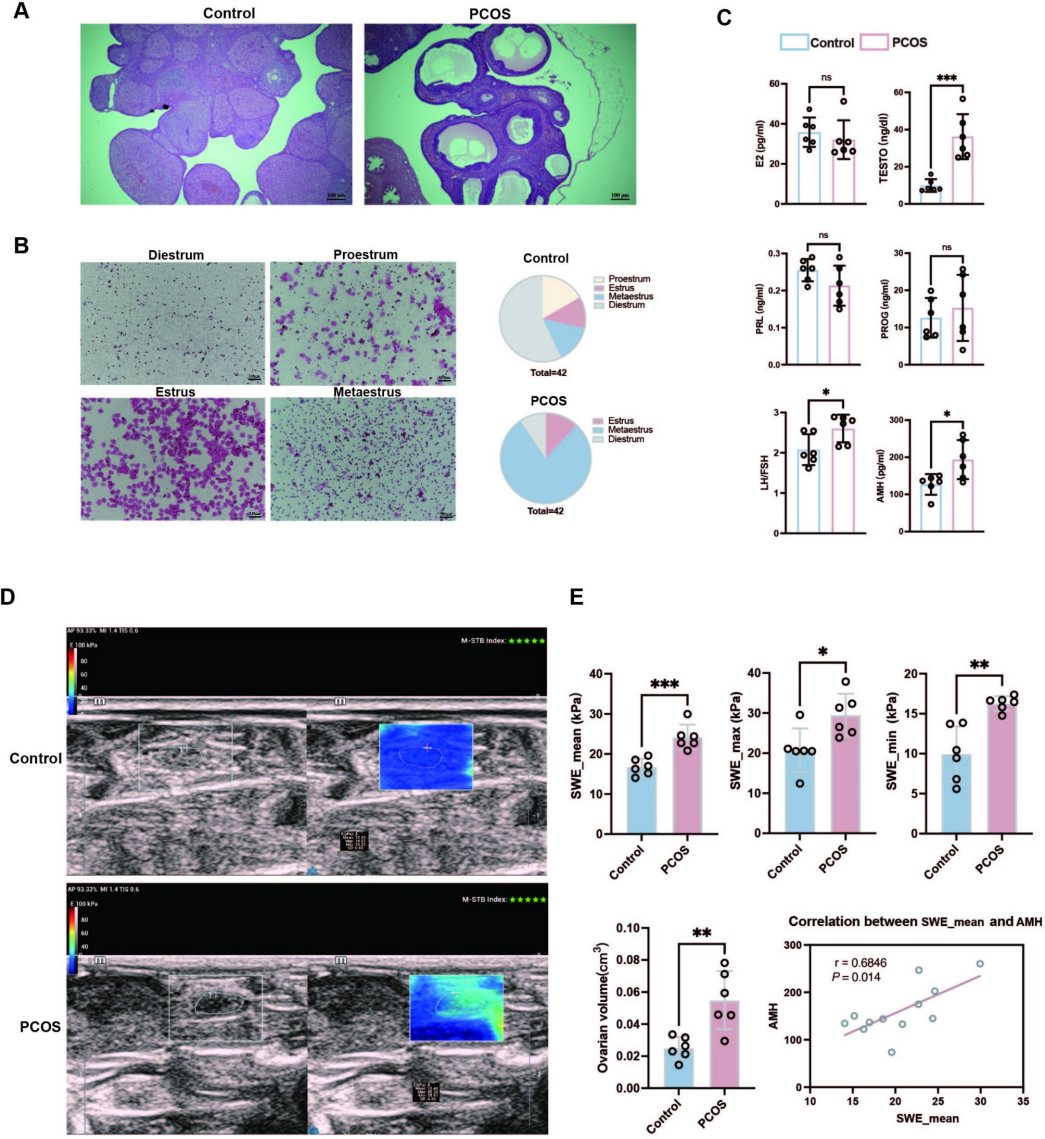
Construction of the DHEA-induced PCOS rat model and measurement of ovarian Young’s modulus and volume. (**A**) HE staining results of ovarian tissues from various groups of rats, magnification: 40×; (**B**) Estrus status of rats in the PCOS and control groups; (**C**) Levels of sex hormones in rat serum; (**D**, **E**) Representative images of ovarian Young’s modulus and volume in both PCOS rats and control rats, along with corresponding histogram statistics. A scatterplot showing the correlation between SWE_ mean and AMH levels in rats. PRGE, progesterone; PRL, prolactin; E2, estradiol, FSH, follicle-stimulating hormone; LH, luteinizing hormone; TESTO, testosterone; AMH, anti-Müllerian tubular hormone; SWE, shear wave elastography. *P < 0.05, **P < 0.01, ***P < 0.001, ns: not significant.

Histological analysis showed that DHEA-treated rats had numerous cystic follicles, reduced corpora lutea, significant estrous cycle disruption, and primarily prolonged estrus (Figs. 4A and B). Subsequently, we assessed the sex hormone levels (PRGE, PRL, E2, LH/FSH, TESTO, and AMH) in rat serum, which revealed significant increases in the TESTO, LH/FSH, and AMH levels in the PCOS group (*P* < 0.05; Fig. 4C) that was consistent with human PCOS, confirming the validity of the model.

We then assessed ovarian stiffness in rats using transabdominal 2D ultrasound and SWE. The ovarian volume, SWE_mean, SWE_max, and SWE_min were significantly higher in the PCOS group than in the control group (*P* < 0.05; Figs. 4D and E). Furthermore, SWE_mean was positively correlated with AMH levels (*r* = 0.6846, *P* = 0.014; Fig. 4E), reinforcing the hypothesis that ovarian stiffness is a key feature of PCOS.

### Increased ovarian tissue stiffness in rats with PCOS may be associated with fibrosis

**Fig. 5:**
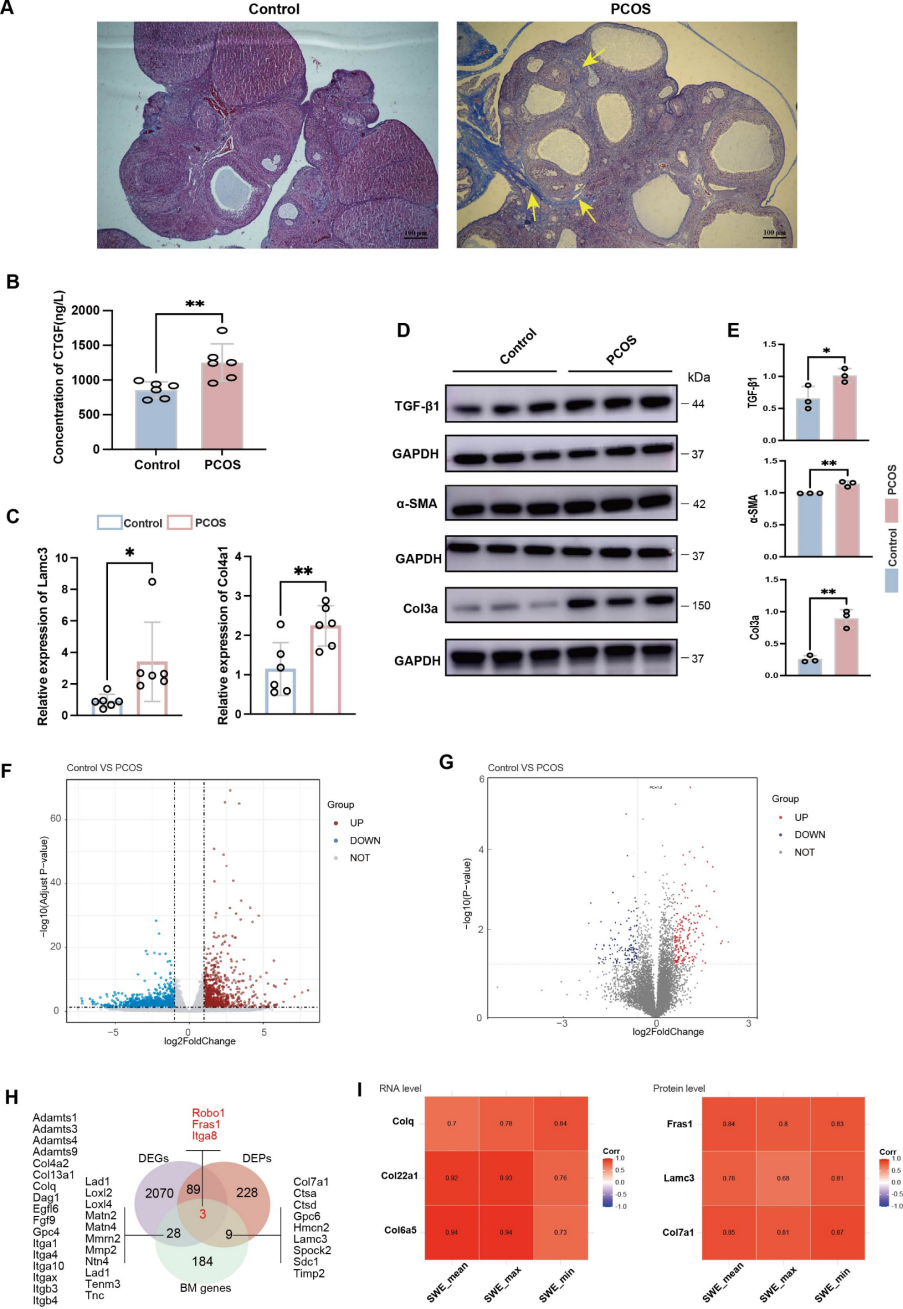
Detecting fibrosis in rat ovarian tissue and combined transcriptional and proteomic analyses. (**A**) Histopathological features of ovarian sections stained with Masson’s stain in the PCOS group demonstrate fibrosis and the deposition of thick collagen fibers, which are stained blue and indicated by yellow arrows (magnification: 40×); (**B**) Serum CTGF levels in PCOS rats were determined by ELISA; (**C**) mRNA levels of Lamc3 and Col4a1 in the ovarian tissue of PCOS rats were quantified by qRT-PCR; (**D**, **E**) Protein levels of TGF-β1, a-SMA and Col3a in the ovarian tissue of PCOS rats were measured by western blot, protein levels displayed in histograms; (**F**, **G**) Volcano plots showing DEGs and DEPs between PCOS and control rat ovarian tissue; (**H**) A Venn diagram showed differentially expressed BM genes/proteins between PCOS and control rat ovarian tissue; (**I**) A correlation heatmap showing the correlation of some of the differentially expressed BM genes/proteins with ovarian elasticity values. DEGs, differentially expressed genes; DEPs, differentially expressed proteins; BM, basement membranes. *P < 0.05, **P < 0.01.

We investigated the increased ovarian stiffness in the rats with PCOS using Masson’s staining, which showed extensive fibrosis in the ovarian medulla due to collagen fiber accumulation (Fig. 5A). The molecular tests revealed higher CTGF levels in the serum and increased mRNA expression of Lamc3 and Col4a1, along with elevated protein levels of TGF-β1, α-SMA, and Col3a in ovarian tissues compared to controls (*P* < 0.05; Figs. 5B–E ). These results suggest that ovarian fibrosis contributes to increased stiffness, as measured by SWE.

To elucidate the molecular basis of ovarian stiffening associated with fibrosis in PCOS, we performed transcriptomic and proteomic analyses of the ovarian tissues from rats with PCOS and controls. We identified a total of 2190 DEGs, while 145,862 peptides and 9717 proteins were identified using the DIA technique, of which 336 DEPs were included in the differential analysis (Figs. 5F and G, and Tables S6 and S7). The dysregulation of the BM genes is considered a key pathogenic factor in fibrosis (Table S8)^19^. We identified 31 and 12 differentially expressed BM genes and proteins, respectively (Fig. 5H). Focusing on several differential BM genes (Colq, Col22a1, and Col6a5) and proteins (Fras1, Lamc3, and Col7a1), we found that their expression correlated with the ovarian Young’s modulus as measured by SWE (Fig. 5I), which suggests that the dysregulation of BM genes may be involved in ovarian fibrosis in PCOS, contributing to increased stiffness.

### Abnormal expression of BM genes in patients with PCOS

**Fig. 6:**
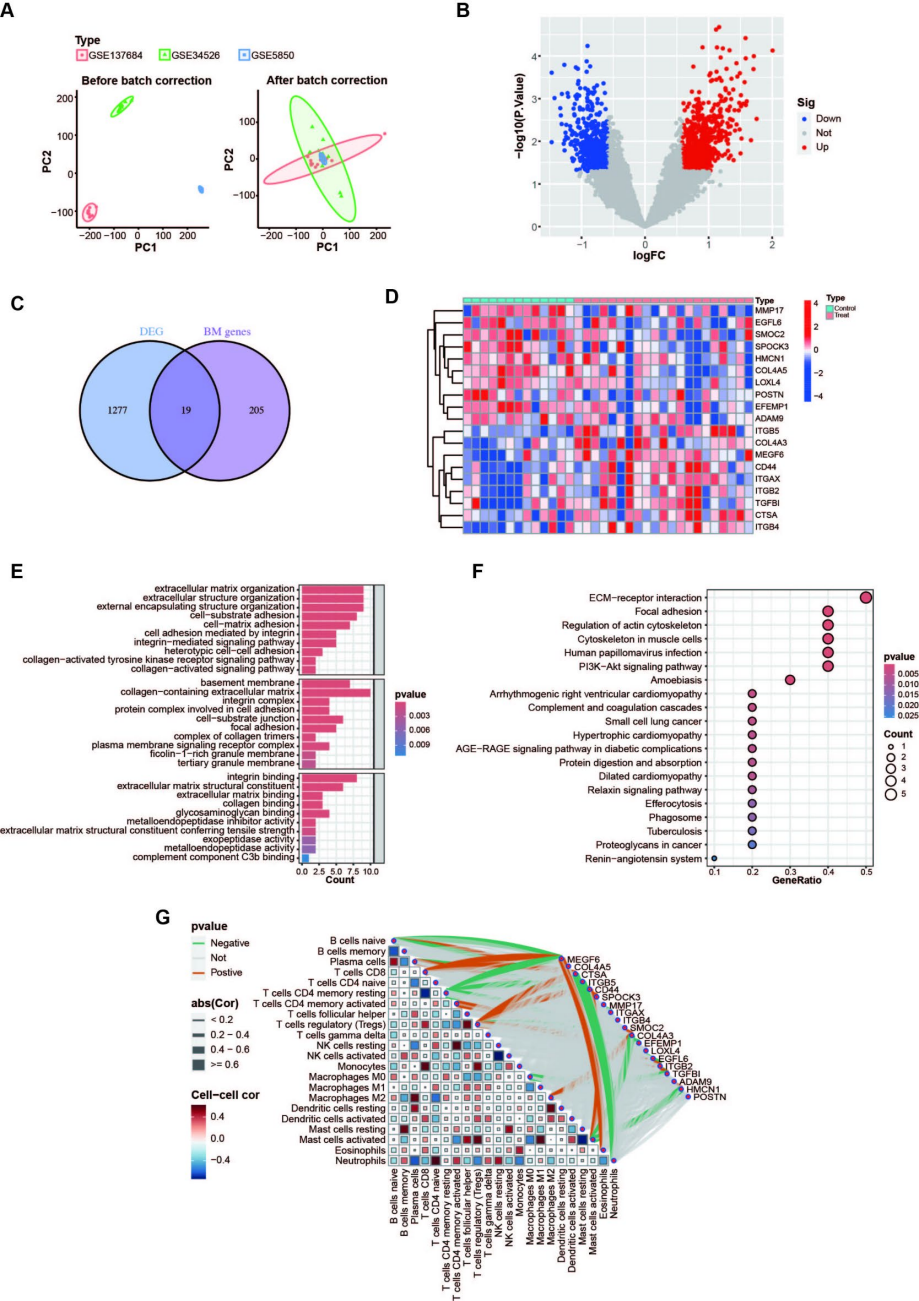
BM genes expression and enrichment analysis in the GEO database for PCOS patients. (**A**) PCA plot of PCOS samples before and after batch correction; (**B**) The volcano plot showing DEGs between PCOS patients and healthy controls; (**C**) Venn diagram showed differentially expressed BM genes between PCOS patients and healthy controls; (**D**) Heat map presented visually differentially expressed BM genes; (**E**, **F**) GO and KEGG enrichment analysis of differentially expressed BM genes; (**G**) Heat map of correlation between hub genes and immune cells, green and red lines indicated positive and negative correlations, respectively, with thicker lines indicating stronger correlations. PCA, principal component analysis. DEGs, differentially expressed genes; BM, basement membranes.

To validate the expression of BM genes in patients with PCOS, we further integrated and analyzed GEO-related datasets using a batch correction approach, thereby comprehensively examining the differences in gene expression between patients with PCOS and healthy control GCs (Fig. 6A). Using the screening criteria of *P* < 0.05 and |logFC| >0.585, we identified 1296 DEGs (Fig. 6B and Table S9). Subsequently, we identified 19 differentially expressed BM genes, a venn diagram and heat maps visually depicted the distinct expression patterns of these BM genes in patients with PCOS compared with healthy controls (Figs. 6C and D). The GO analysis showed that the BM genes were mainly enriched in terms of extracellular matrix (ECM) organization, extracellular structure organization, BM, collagen-containing ECM, and integrin binding (Fig. 6E). The KEGG analysis showed that BM genes were mainly enriched in ECM-receptor interaction, focal adhesion, regulation of the actin cytoskeleton, cytoskeleton in muscle cells, human papillomavirus infection, and the PI3K-Akt signaling pathway (Fig. 6F). The immune infiltration analysis was performed using the CIBERSORT algorithm to predict the presence of infiltrating immune cells near GCs. By evaluating the correlation between BM genes and immune cells, we discovered that BM genes associated with PCOS were positively or negatively linked to specific immune-infiltrating cells (Fig. 6G and Table S10). These results indicate that BM genes not only play a key role in the altered structural organization and stiffness of the ovarian ECM in patients with PCOS but may also influence the development of fibrosis in PCOS by participating in the regulation of the immune microenvironment.

## Discussion

Our study evaluated increased ovarian stiffness in patients with PCOS using the transvaginal SWE technique and determined its diagnostic value. By constructing a rat model of PCOS, we conducted SWE examinations and combined multi-omics analyses to explore the association between increased stiffness and fibrosis, which may be attributed to altered gene expression in the BM. These results demonstrate the potential of SWE as a diagnostic tool for PCOS and provide new insights into the molecular mechanisms underlying ovarian stiffening.

We used transvaginal SWE to detect elevated ovarian elasticity and hardening in patients with PCOS, which was similar to previous studies^13,14,20^. However, some studies have reported no significant differences in ovarian elasticity values between patients with PCOS and healthy controls using SWE^21,22^. These studies used transabdominal SWE, which we believe may have been influenced by abdominal adiposity, potentially introducing confounding factors to the results. In our study, we did observe a significant difference in ovarian stiffness, which may be attributed to the more precise and localized measurement of transvaginal SWE than transabdominal SWE. Transvaginal SWE enables direct imaging of ovarian tissue, avoiding interference from abdominal fat, which may mask slight alterations in tissue elasticity. We also recognize that factors such as the stage of the PCOS progression, differences in hormone therapy, and patient heterogeneity may affect the results. Some studies may include patients undergoing treatments that may have a modifying effect on ovarian stiffness. In contrast, our study selected patients with more defined clinical characteristics. In addition, methodological factors such as sample size as well as the timing of SWE measurements may all affect the properties of ovarian tissue, leading to influencing the study results.

We considered the number of follicles, ovarian volume, and SWE_mean to be independent risk factors for PCOS in our regression analysis. An increased number of follicles reflects excessive androgen production and follicular stagnation associated with hyperinsulinemia and insulin resistance^23^. We hypothesized that an increase in the number of follicles may increase intraovarian tension and consequently lead to a change in ovarian stiffness. However, this relationship needs to be supported by more direct evidence. We were greatly inspired by studies that utilized atomic force microscopy (AFM) to map and quantify the mechanical microenvironment within normal mouse ovaries at high resolution^24^. Our next step will be to perform AFM on rat ovarian tissues to obtain direct measurement of the Young’s modulus of SWE for detecting changes in the mechanical environment of the ovary in PCOS. We observed that SWE_mean was closely associated with AMH levels, which is a marker of the ovarian reserve^25^, suggesting that SWE may serve as a potential surrogate marker for ovarian fibrosis and reserve dysfunction.

To the best of our knowledge, this is the first study to apply SWE to tests involving a rat model of PCOS. In addition, this is the first study to directly investigate the histological and molecular bases of changes in SWE measurements. The PCOS rat model exhibited significant ovarian fibrosis, highlighting the critical role of fibrosis in this pathology, which is characterized by excessive deposition of collagen and other ECM components^26–28^. Fibrosis may be driven by chronic low-grade inflammation and oxidative stress, which contribute to abnormal follicular development, impaired hormone secretion, and ovarian dysfunction, all of which are key features of PCOS^29–31^. Multiomics analyses have highlighted alterations in BM genes, such as laminin, collagen, and integrins, which are vital components of the ECM. They affect cell polarization, adhesion, migration, and differentiation, and their dysregulation is associated with various fibrotic disorders^32–36^. We observed genetic alterations in BMs at both the RNA and protein levels, which were associated with ovarian stiffness. This finding is consistent with those of previous studies indicating that ECM remodeling is dysregulated in PCOS, which can lead to ovarian dysfunction and fibrosis^37^. Furthermore, this study verified the alterations in BM genes in patients with PCOS using the GEO database. The roles and mechanisms of these genes in PCOS development were elucidated using bioinformatics analyses. We conclude that alterations in BM-related genes affect functions and pathways, such as integrin binding and ECM-receptor interactions, and are associated with immune infiltration, ultimately contributing to ovarian fibrosis in PCOS. SWE technology responds to these molecular landscapes to a certain degree.

This study contributes significantly to our understanding of PCOS. SWE has greatly improved PCOS diagnosis by providing information on ovarian stiffness and complementing traditional ultrasound morphology and blood flow characteristics. This study established a correlation between ovarian stiffness and molecular changes in BM genes, reinforcing the biological relevance of SWE technology and paving the way for targeted therapies focused on ECM components or signaling pathways. It also employed a multifaceted approach, integrating ultrasound imaging, histology, and molecular and bioinformatics tools, demonstrating comprehensive profiling, from imaging to molecular insights.

Nevertheless, some limitations were of course noted. The sample size of future studies should be larger and include multicenter data collection. Moreover, a prospective study should be conducted to validate the generalizability of this technique for diagnosing PCOS, and to establish its practicality in routine clinical practice. Establishing a direct association analysis of human tissue samples using this technique would help illustrate the biological basis of the technique more intuitively. This study showed that changes in BM genes may mediate ovarian fibrosis through ECM pathways, thereby increasing ovarian stiffness; hence, future research should clarify the roles of these genes in ovarian tissues and their effects on structure and function using knockout or overexpression models.

## Conclusion

Our results support the value of SWE in the diagnosis of PCOS and suggest that increased ovarian stiffness may be associated with altered BM genes that mediate ovarian fibrosis in patients with PCOS. Further investigation is warranted to fully elucidate the functional mechanisms of these genes and validate the wide application of SWE in clinical practice.

## Electronic supplementary material

Below is the link to the electronic supplementary material.

Supplementary Material 1

Supplementary Material 2

Supplementary Material 3

## Data Availability

The data generated in this study are available within the paper. Derived data supporting the findings of this study are available upon reasonable requests from the corresponding author.
