# Supplementary material for: Evaluation of ovarian stiffness and its biological mechanism using shear wave elastography in polycystic ovary syndrome
Source: Sci Rep. 2025 Jan 2;15:585. doi: 10.1038/s41598-024-84338-8 (PMC11695736; doi:10.1038/s41598-024-84338-8)
Supplement: Supplementary file 1 — Supplementary Material 1 [file 41598_2024_84338_MOESM1_ESM.docx]

**SUPPLEMENTARY METHODS**

**Ovarian histochemical staining and vaginal smears**

Rat ovary specimens were fixed and embedded in paraffin. The thickness of ovarian paraffin sections was 4 μm. the sections were stained with HE staining kit (Beijing Solarbio Science and Technology Co., Ltd, Beijing, China) to observe the pathological changes in ovarian structure and morphology and Masson Trichrome Stain Kit (Beijing Solarbio Science and Technology Co., Ltd, Beijing, China) to stain collagen. The estrous cycle stages, including proestrus (P), estrus (E), metestrus (M), and diestrus (D), were identified by analyzing three types of vaginal epithelial cells. Vaginal secretions were spread on glass slides and subsequently stained using the HE kit.

**Analysis of serum samples**

Serum levels of Connective Tissue Growth Factor (CTGF) and sex hormones progesterone (PRGE), prolactin (PRL), estradiol (E2), follicle-stimulating hormone (FSH), luteinizing hormone (LH), testosterone (TESTO), and anti-Müllerian tubular hormone (AMH) were measured using enzyme-linked immunosorbent assay kits (MeiMian Biotech Co., Ltd, Jiangsu, China) according to the methods provided by the manufacturer.

**qRT-PCR analysis**

The mRNA expression levels of Lamc3 and Col4a1 in ovarian tissues were detected by qRT-PCR. Total RNA was purified using the RNA-quick Purification Kit (ESscience Biotech Co., Ltd, Shanghai, China), and the concentration and purity of the RNA were determined using a nucleic acid protein detector (Applied Biosystems, Carlsbad, USA). Subsequently, reverse transcription was performed using the PrimeScriptTM RT Reagent Kit with gDNA Eraser (TaKaRa Bio Inc., Tokyo, Japan). qRT-PCR was conducted using the TB Green® Premix Ex Taq™ (Tli RNaseH Plus) and 10 μM primer (final concentration) with SYBR, following the manufacturer's instructions (TaKaRa).The primer sequences for the target genes were synthesized by Sangon Biotechnology Co., Ltd. ( **Table S1**), and the expression of the target genes was normalized to the GAPDH gene using the 2-ΔΔCT-method. Each reaction was carried out in triplicate.

**Western Blot experiments**

Proteins were extracted by centrifugation using RIPA lysis buffer. The protein concentration was measured using the microbicinchoninic acid method. 20 μg of each sample was separated on a 4-20% SDS-PAGE gel and transferred to a polyvinylidene fluoride (PVDF) membrane (Millipore, Billerica, USA)(0.45 um) using standard procedures.The membrane was blocked with 5% skim milk in TBST buffer with Tween 20 at room temperature for 2 hours, followed by overnight incubation with the primary antibody (GAPDH, 1:30,000, AF7021, Affinity, Jiangsu, China; TGF-β1, 1:2,000, HA721143, HUABIO, Hangzhou, China; α-Smooth Muscle Actin (α-SMA),1:10,000, ET1607-53,HUABIO; Col3a, 1:2,000, HA720050, HUABIO) at 4°C. After incubation with a secondary antibody, goat anti-mouse IgG (H+L)-horseradish peroxidase (S0002, 1:10,000, Affinity) at room temperature for 1 h. Bands were photographed and developed using ECL (Peiqing JS-1070 Chemiluminescence Imaging System, Shanghai, China). Specific bands were quantified using ImageJ software (NIH, Bethesda, MD, USA).

**RNA-sequencing (RNA-seq)**

The NEBNext Ultra RNA Library Prep Kit for Illumina (NEB, USA) was employed to generate sequencing libraries, following the manufacturer's instructions. Index codes were incorporated to distinguish sequences from each sample. PCR products were purified using the AMPure XP system, and library quality was assessed on an Agilent Bioanalyzer 2100 system.

**DIA proteomics**

The resulting peptides were desalted, concentrated, and reconstituted in 40 µl of 0.1% formic acid. Peptide content was quantified by UV absorbance at 280 nm. DIA analysis was performed using a nanoliter flow rate Vanquish Neo system (Thermo Fisher Scientific) for chromatographic separation, and samples separated by nanoliter HPLC were analyzed by DIA (data-independent mass spectrometry) using an Astral Astral high- resolution mass spectrometer (Thermo Scientific). The samples were separated by nanoscale HPLC and analyzed by DIA (data-independent) mass spectrometry using an Astral high-resolution mass spectrometer (Thermo Scientific).

The detection mode: positive ions, the scanning range of parent ions was 380-980 m/z, the resolution of primary mass spectrometry was 240000 at 200 m/z, the Normalized AGC Target was 500%, and the Maximum IT was 5 ms. The DIA data acquisition mode was used in the MS2, and 299 scanning windows were set, and the Isolation Window was set. MS2 used DIA data acquisition mode, set 299 scanning windows, Isolation Window at 2m/z, HCD Collision Energy at 25ev, Normalized AGC Target at 500%, Maximum IT at 3ms.

**Bioinformatics analysis**

The GEO datasets GSE137684, GSE34526, and GSE5850 were combined into a unified dataset. To mitigate batch effects in subsequent analyses, the ComBat algorithm from the R package sva was utilized. This method is commonly used to harmonize gene expression data by adjusting values according to known batch information, thereby eliminating batch-related biases.

In this study, the *limma* R package was utilized to identify DEGs between PCOS patients and controls. The criteria for determining DEGs were set as follows: a P-value threshold of less than 0.05 and a log fold change greater than 0.585. The ggplot2R software package was used to map the volcano, and heat maps were used to indicate the up-regulation and down-regulation of differential BMs genes.

To assess the functional implications of the differential BMs genes, GO functional analyses were conducted using the *clusterProfiler* R package. These analyses aimed to evaluate the BMs genes in terms of their involvement in biological processes (BP), cellular components (CC), and molecular functions (MF). Additionally, KEGG pathway analyses were performed using the same R package. KEGG pathway analyses identified significant enrichment of biological functions associated with a P-value threshold of less than 0.05. To account for multiple testing, the false discovery rate-adjusted P-value, referred to as the Q-value, was calculated. For the GO analyses, a Q-value cutoff of less than 0.05 was used to determine the significantly enriched functional categories.

Immune infiltration estimations via CIBERSORT

This analysis aimed to evaluate the relationship between the expression of BMs genes and immune-related functions. Furthermore, CIBERSORT, a gene expression-based deconvolution algorithm that evaluates immune cell infiltration signatures, was utilized to examine immune infiltration patterns in PCOS samples. By determining the proportions of different immune cell types, the links between differential BMs genes and specific immune cell populations were investigated. Spearman correlation coefficients, computed using the R statistical package, were used to assess the associations between differential BMs genes and infiltrating immune cells. The interaction outcomes were presented visually with the *ggplot2* package in R.
