# Supplementary figures and images for: Evaluation of ovarian stiffness and its biological mechanism using shear wave elastography in polycystic ovary syndrome
Source: Sci Rep. 2025 Jan 2;15:585. doi: 10.1038/s41598-024-84338-8 (PMC11695736; doi:10.1038/s41598-024-84338-8)

Figure 5D

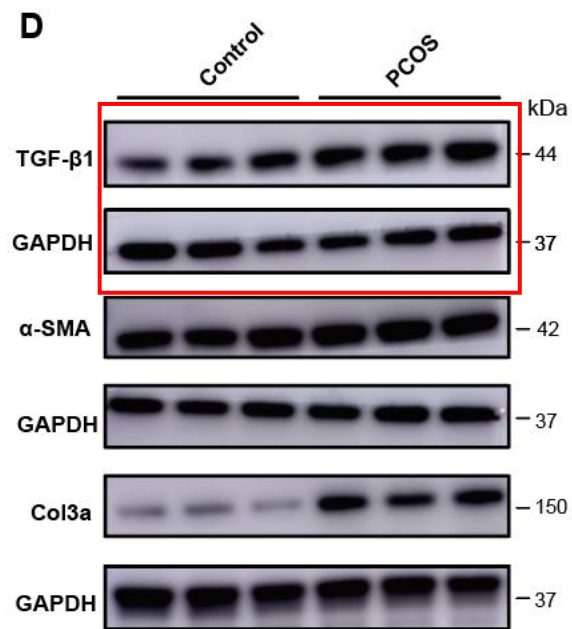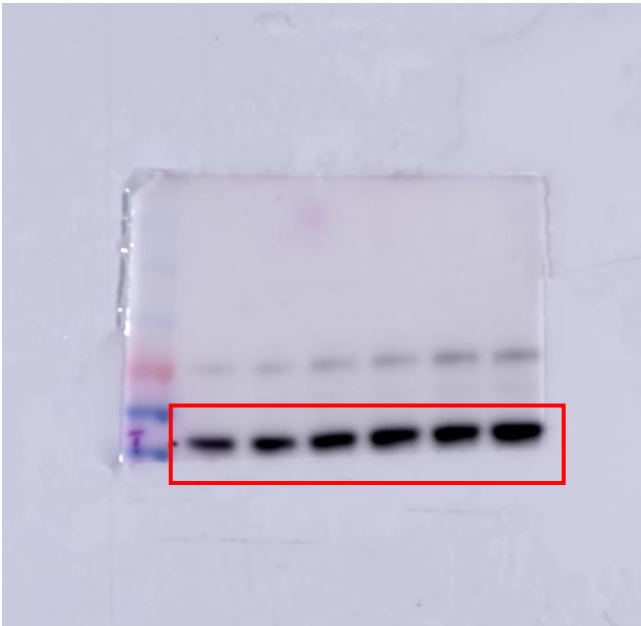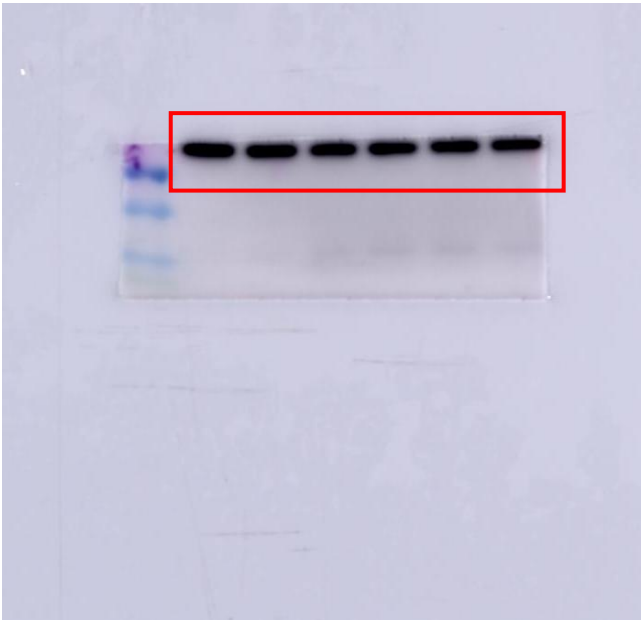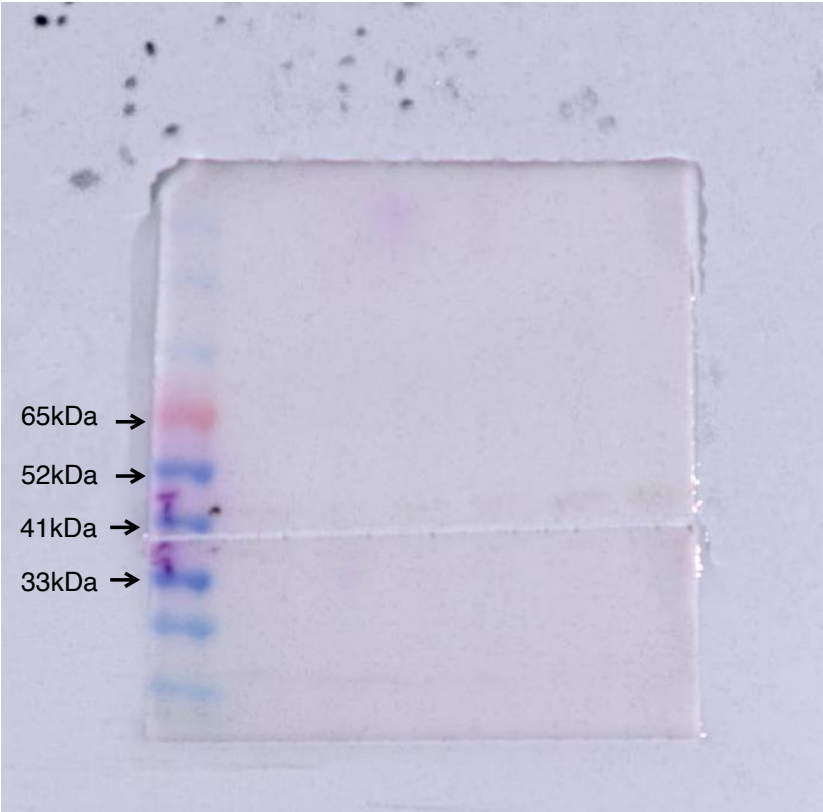

Figure 5D

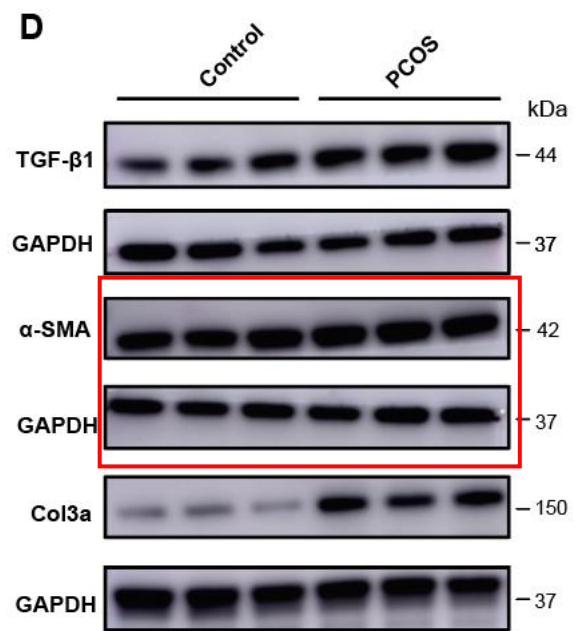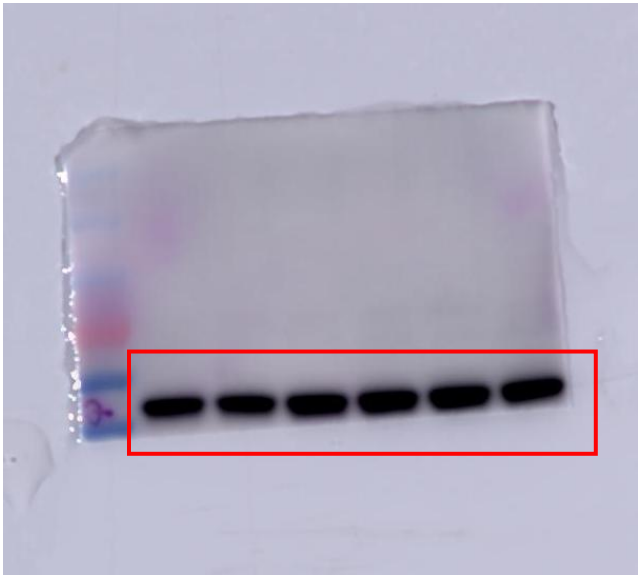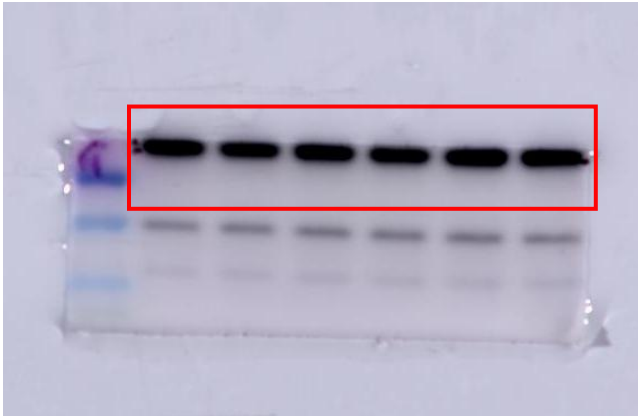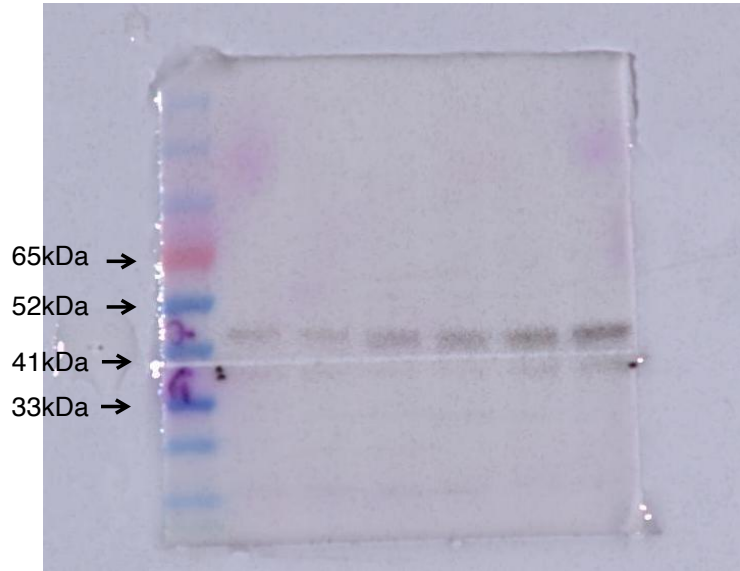

Figure 5D

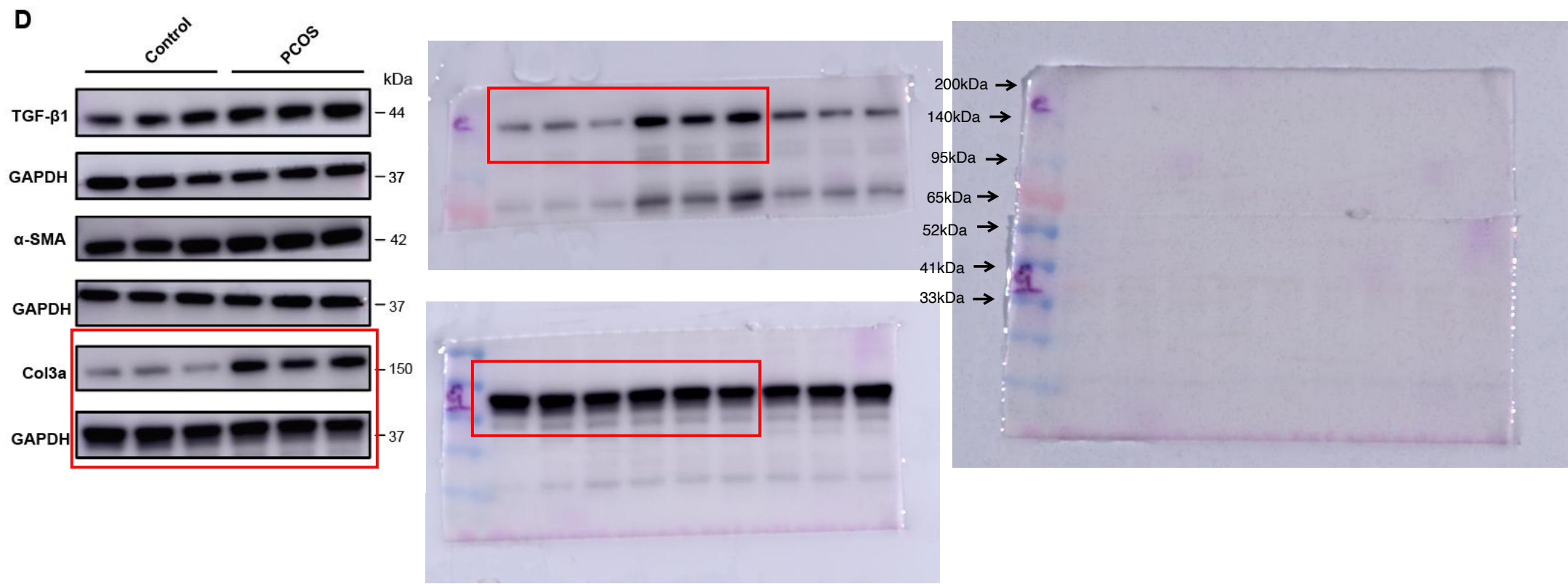

Supplement: Supplementary file 2 — Supplementary Material 2 [file 41598_2024_84338_MOESM2_ESM.pdf]
